# Real-Time Stress Level Feedback from Raw Ecg Signals for Personalised, Context-Aware Applications Using Lightweight Convolutional Neural Network Architectures

**DOI:** 10.3390/s21237802

**Published:** 2021-11-24

**Authors:** Konstantinos Tzevelekakis, Zinovia Stefanidi, George Margetis

**Affiliations:** Foundation for Research and Technology—Hellas (FORTH), Institute of Computer Science, GR-70013 Heraklion, Greece; ktzevel@ics.forth.gr (K.T.); zinastef@ics.forth.gr (Z.S.)

**Keywords:** stress assessment, convolutional neural network, ECG signal, real time, sliding window

## Abstract

Human stress is intricately linked with mental processes such as decision making. Public protection practitioners, including Law Enforcement Agents (LEAs), are forced to make difficult decisions during high-pressure operations, under strenuous circumstances. In this respect, systems and applications that assist such practitioners to take decisions, are increasingly incorporating user stress level information for their development, adaptation, and evaluation. To that end, our goal is to accurately detect and classify the level of acute, short-term stress, in real time, for the development of personalized, context-aware solutions for LEAs. Deep Neural Networks (DNNs), and in particular Convolutional Neural Networks (CNNs), have been gaining traction in the field of stress analysis, exhibiting promising results. Furthermore, the electrocardiogram (ECG) signals, have also been widely adopted for estimating levels of stress. In this work, we propose two CNN architectures for the stress detection and 3-level (low, moderate, high) stress classification tasks, using ultra short-term raw ECG signals (3 s). One architecture is simple and with a low memory footprint, suitable for running in wearable edge-computing nodes, and the other is able to learn more complex features, having more trainable parameters. The models were trained on the two publicly available stress classification datasets, after applying pre-processing techniques, such as data pruning, down-sampling, and data augmentation, using a sliding window approach. After hyperparameter tuning, using 4-fold cross-validation, the evaluation on the test set demonstrated state-of-the-art accuracy both on the 3- and 2-level stress classification task using the DriveDB dataset, reporting an accuracy of 83.55% and 98.77% respectively.

## 1. Introduction

Chronic stress poses a risk factor for serious health conditions and episodes, such as hypertension, heart attack, and stroke. Apart from the grave long-term consequences, short-term stress can also affect behavior and mental processes such as decision making, which is of paramount importance in a diversity of application domains (e.g., automotive, aviation, public protection and disaster relief, etc.). Researchers and developers of personalised, context-aware applications for public protection practitioners are increasingly acknowledging the need for leveraging user stress level information, to cater for the varying requirements depending on its level. In this respect, DARLENE, a European Union funded project [1] aims at providing technologies that enable law enforcement agents (LEAs) and in general first responders to make more informed and rapid decisions, especially in situations where time is of the essence. Provided that LEAs’ performance and awareness of the situation are directly influenced by their stress level, a system is being developed that takes this into account by providing them with contextual, real-time information, specific to their mission, through worn Augmented Reality glasses. In particular, a supporting sub-system, deployable to edge devices, is being implemented, which classifies the level of acute, short-term stress in real time.

With respect to assessing and inferring the stress state and the cognitive load of an individual, many research efforts have been conducted. Stress assessment is traditionally based on questionnaires where the subjects fill in their stress level rating on some scale. Such methods are the Relative Stress Scale (RSS) [2], Fear Survey Schedule (FSS) [3] and Cook-Medley Hostility Scale [4]. These methods, by definition, constitute a subjective way of measuring stress and can also be conducted as self-assessments without the need of experts [2,5]. Another approach to stress assessment is to collect and measure stress-related hormones, such as cortisol and catecholamine [6]. However, these approaches are not suitable for some applications where the subject’s concentration must remain intact and any distraction should be avoided.

Thus, automated and non-invasive procedures that are mostly based on physiological signals (e.g., electrocardiogram (ECG) electrodermal activity (EDA), the electrical activity of the scalp (EEG)) deem to be more suitable for this kind of applications [7]. To that end, a variety of machine learning models have been developed to automatically assess stress, based on data that can be collected in an unobtrusive manner [8,9].

The analysis of the electrical activity of the heart (ECG) [10], the electrodermal activity (EDA) [11], the electrical activity of the scalp (EEG) [12] and others [13] can be used to assess the mental stress of an individual both robustly and unobtrusively during stressful and demanding tasks [7,14].

**Figure 1:**
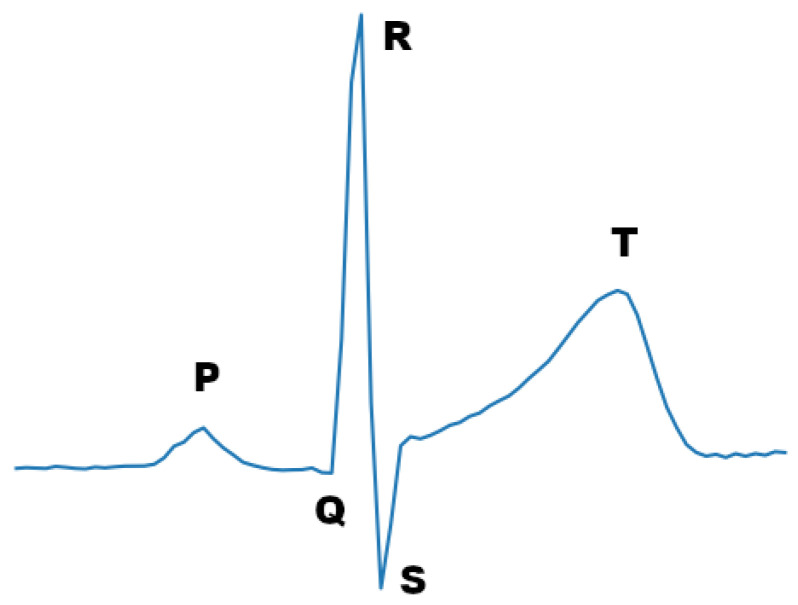
ECG signal period.

One of the most prevalent physiological signals that is used today for this kind of task is the ECG signal. The ECG signal reflects the electrical heart activity by detecting changes in the voltage on the surface of the skin due to the electrical activity of the heart. ECG sensors are made to be highly sensitive to electrical activity and capture the main electrical signals that are produced by cardiac cells depolarizing and re-polarizing. The most dominant waveform, in terms of amplitude, which is formed by this activity is the QRS waveform, also known as QRS complex (see Figure 1). To capture this wave, using three electrodes, the bipolar limb lead system can be used. Each lead configuration, specified by the Einthoven’s Triangle, results in a different ECG signal morphology. For instance, an ECG signal with sharp R-waves can be acquired by placing the sensors in either lead II or lead III configuration [15].

Stress classification tasks that use primary input ECG signals have been well researched. The ECG signal, as a physiological measure, has been found by a lot of researchers to be a reliable measure that one can use to tackle the problem of stress classification [13,16,17]. One of the main reasons is that a direct influence of both the sympathetic and parasympathetic nervous system exists in the ECG signal [18], where the integrated response is a consequence of their energy balance [19]. Specifically, heart rate variability (HRV), which is a feature extracted from the ECG signal, is able to index the cardiac vagal tone, which represents the contribution of the parasympathetic nervous system to cardiac regulation and it is known to be relevant with many phenomena relevant for psychophysiological research [17].

In this work, we aim to tackle the stress classification task, both for 2 and 3 levels of stress, using raw ECG signals. Our work includes the data pre-processing and data augmentation of open-access stress related datasets as well as the design and implementation of Deep Neural Network (DNN) architectures. In particular, we have assessed the data quality of these datasets and have also elaborated on potential problems and limitations. Furthermore, we have implemented two DNN architectures, based on well established approaches, one that is simple and memory efficient, enabling edge device deployment, and one that is deeper and has the capacity of learning complex features. The resulting models were trained and validated both on the *Electrocardiogram, skin conductance, and respiration from spider-fearful individuals watching spider video clips* (Arachnophobia) [20] and on the *Stress Recognition in Automobile Drivers dataset* (DriveDB) [14], achieving state-of-the-art performance in comparison to similar DNN approaches.

## 2. Related Work

### 2.1. Conventional Machine Learning Approaches

A conventional approach for ECG signal classification tasks based on machine learning (ML) approaches is to use shallow models that take as input manually constructed features. The most common algorithms that are used in the stress classification task are Logistic Regression (LR) [21,22], Support Vector Machines (SVM) [23,24], Random Forests (RF) [25], Bayesian Networks (BN) [26], and K-Nearest Neighbors (KNN) [27]. In addition, in order to boost the overall performance, researchers frequently use hybrid techniques or model ensembles.

The most frequent type of features that these models take as input is HRV related features. These features relate to the standard deviation of heart periods within the recording epoch [18] and they are considered as appropriate measures of both heart’s short-term and long-term variation regarding ECG signals [28]. Other features that these models take as input can be found in [17,22].

### 2.2. Deep Learning Approaches

In traditional ML approaches, the features are manually engineered by field experts. On the other hand, provided that the classification task is compositional and the dataset size is sufficient, DNNs have the ability to automatically learn useful features from the data. Furthermore, in many cases, these features outperform handcrafted ones, leading to improved classification accuracy. A concrete example can be derived from the computer vision discipline where the dominance of the deep convolutional neural networks is apparent. CNNs leverage three important principles, namely sparse interactions, parameter sharing, and equivariant representations. These principles result in a reduced number of parameters and the computation of local features that can be incorporated to effectively classify a sample. When they are combined with pooling layers, they can also compute representation invariant features [29]. In addition, one-dimensional CNNs offer these properties in a lower complexity [30], and thus they are suitable for several 1D signal processing tasks. Finally, Recurrent Neural Networks (RNNs) are also being used with great success, due to the sequential nature of the signals. In particular, they are used in combination with convolutional layers that downsample the signal in order to reduce the size of the RNN input sequence.

Towards this direction, He et al. [31] demonstrated the power of DNNs and specifically CNNs for the stress classification task, based on ECG signals. This was done by comparing the performance of a CNN against conventional HRV-based methods for stress classification, acquiring a significantly better accuracy in the former case. Moreover, Hwang et al. introduced Deep ECGNet [32], exemplifying that there is no need for a very deep network to accomplish high accuracy on the task. In addition, they showed that it is essential, for the performance of the model, to exploit features of the ECG signal by taking advantage of specific characteristics of the signal’s morphology. In particular, they set the kernel size to span a complete ECG period, on average. To avoid the peak phase difference problem, they used max-pooling operation, which guarantees that, at a specific pooling size, it can extract the peaks regardless of the peak point position. Similar to their approach, our models constitute a reduced number of layers, with respect to the related literature. Moreover, we adopted their kernel size and pooling size, in one of our models. Another work that utilizes a 1D CNN, based on the ECG signal, is DeepERNet [33]. However, it also combines the respiration (RSP) signal of the subject, which can be measured as the rate or volume of air exchange.

On the other hand, instead of using the one-dimensional input of the raw ECG measurements, other approaches transform the signal to two dimensional images that they later feed to 2D CNN models [34,35]. Kang et al. [36] have also used similar methods to transform the signal to two dimensions, both in frequency and time, adding a Long-Short Term Memory (LSTM) unit to their architecture so as to be able to exploit the temporal features.

### 2.3. Available Stress Datasets

Although limited in number, some publicly available ECG stress-related datasets exist and can be used to train ML models.

The DriveDB [14] contains a collection of multiparameter recordings from healthy volunteers, taken while they were driving on a prescribed route including city streets and highways in and around Boston, Massachusetts. The objective of the study for which these data were collected was to investigate the feasibility of automated recognition of stress on the basis of the recorded signals, which include ECG, QRS (right trapezius), and GSR (galvanic skin resistance) signals measured on the hand and foot, as well as respiration data.

The Arachnophobia [20] dataset contains ECG, GSR, and respiration measures as raw data (unfiltered, unprocessed) recorded from consented, spider-fearful individuals with the sampling rate set to 100 Hz per channel having a 10-bit resolution. Specifically, 80 spider-fearful individuals aged between 18 and 40 years were exposed to several clips that were all taken from television (TV) documentaries showing detailed shots of spiders. The main focus of this randomized controlled trial was to investigate if the use of an HRV biofeedback intervention could be a promising therapeutic add-on to exposure therapy for specific phobias.

The two datasets above include the necessary information and provide a methodology to annotate the data with three stress level labels (low, moderate, high) and they are both part of the Physionet research resource  [37]. Additional public datasets for stress classification are the Wearable Stress and Affect Detection (WESAD) dataset [38] and the SWELL Knowledge Work dataset for Stress and User Modeling Research [39]. However, they do not consist of annotated ECG signals for 3-level stress classification, and were therefore excluded from this study.

## 3. Methods

Two deep convolutional neural networks have been developed towards classifying raw ultra-short ECG signals (3 seconds) to 2 (no stress, stress) and 3 classes (low, moderate, high). The architectures were trained and validated using two pertinent publicly available datasets, namely DriveDB and Arachnophobia. In the following sub-sections the overall process of dataset pre-processing, model training, and evaluation is discussed.

### 3.1. Dataset Pre-Preprocessing

The datasets used in this work are the DriveDB and the Arachnophobia. To the best of our knowledge, they are the only publicly available datasets that include annotated ECG signals with three stress states (low, moderate, and high stress). Both follow the lead II standard configuration to capture the ECG signal, which results in minimizing the motion artifacts and producing a rhythm trace with sharp R-waves.

Since the datasets do not directly provide the samples together with their corresponding labels, they need to be converted to a label-sample form, following a dataset-specific procedure. In the case of DriveDB, this procedure entails leveraging the marker signals that were embedded in the ECG signals by the authors. The marker signal has distinguishable peaks that separate the ECG signal segments, which are annotated with different stress states. In the case of the Arachnophobia dataset, the annotation information was not provided explicitly. In particular, the authors provide two annotation methodologies that can be used, which leverage the observed relation between beats per minute (bpm) and stress state, as described in detail in their paper [20]. The procedure followed for each dataset is detailed in the next subsections, Section 3.1.1 and Section 3.1.2.

#### 3.1.1. DriveDB

This dataset provides data from 17 subjects that were recorded while they were driving on a prescribed route including city streets and highways in and around Boston, Massachusetts. In this work, we focus only on the ECG and marker signal data. To parse and resample the data we used the waveform database (WFDB) software package [37]. To apply the annotations indicated by the marker signal, which was initially sampled at the frequency of 15.5 Hz, we first had to upsample it to match the frequency of the ECG signal (496 Hz) and then to locate its peaks. This was done with the find peaks method of the SciPy python package [40]. Moreover, to acquire results from both datasets at the same sampling frequency, in some of our experiments, we down-sampled the ECG signal of the DriveDB dataset to match the sampling frequency of Arachnophobia (i.e., 100 Hz).

**Figure 2:**
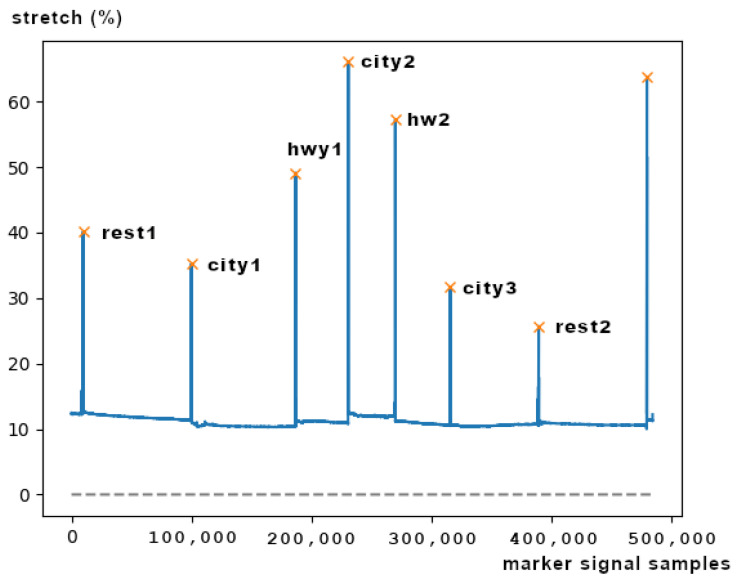
DriveDB driving event segments and peaks of the marker signal, created using a respiration sensor.

As illustrated in Figure 2, the seven segments that are indicated from the 8 peaks correspond to the seven driving events that are annotated with the corresponding stress state. These events are respectively the initial resting phase (rest1), city road driving period 1 (city1), highway driving period 1 (hwy1), city2, hwy2, city3, and the final resting phase (rest2). The data of the other driving events of the study were not included in the resulting dataset. Due to the difficulty of those experiments, there were several errors and problems with the ECG and marker signals of some subjects. Table 1 summarizes the comments and the observations we have made during the dataset inspection.

As a consequence, the number of the drives that were included in the resulting dataset were 9. The duration of the 7 driving events per driver, in minutes, have already been calculated by Akbas et al. [41]. The minimum event duration is 5.12 min; thus, as the dataset authors did [14,42], we partitioned the data into segments of 5 min durations, so as to create a dataset that is equally biased from each subject and to avoid any marginal signal noise that is located at the borders of each segment. Moreover, the annotation of the driving events was based on the task; for instance, city-driving was considered more stressful than highway-driving. The task-based assumptions were also supported by questionnaires. Table 2 illustrates the driving events and the corresponding annotations for two and three classes of stress, respectively.

#### 3.1.2. Arachnophobia

This dataset contains data recorded from spider-fearful individuals while they were watching clips from documentaries that contained frames depicting spiders. The available data result from 64 subjects. The data are organized in folders, one for each subject. Each folder has the data from the sensor recordings specific to the subject. Although this dataset does not provide a marker signal, for each subject, it provides a file (triggers.txt) that includes timestamps for each clip. So, if the annotation per clip is known, the correct label can be assigned to the data corresponding to these clips later. A methodology to annotate the clips has been provided in the dataset’s paper [20]. The authors propose two approaches that can be followed, namely the HR and EDA approach and the SB approach. The latter cannot be followed because it is based on the subjective arousal ratings of the subjects, which are not available in the dataset. The former leverages the assumed relation between stress and the two physiological signals. This approach is subdivided in clip-based and subject-based approaches. The clip-based approach establishes labels to the video clips by sorting the average of the normalized signal of all the records. On the contrary, the subject-based approach establishes labels to the video clips by considering individual responses such that the clips were annotated using the individual normalized mean for HR and EDA. In our work, we followed the subject based approach and we used only the ECG signal which was recorded at 100 Hz.

#### 3.1.3. ECG Signal Sample Fragmentation

In accordance with similar DNN approaches, each ECG signal segment corresponding to a stress level was further fragmented to time windows. The trade-off for the selection of window size is information versus inference time. As the window size increases, the more RR intervals (see Figure 1) it contains, which can be leveraged to establish a more accurate prediction of the stress level. However, by expanding the window size not only do we increase the inference latency, due to extra computational cost, but we also allocate more memory resources. In this work, considering the requirement for our model to be deployable on edge devices, we selected a window size of 3 s.

Table 3 summarizes the number of samples per dataset and per class, using a window size of 3 s. We can observe that the class distribution is slightly unbalanced, with both datasets having fewer samples in the ‘low stress’ class.

#### 3.1.4. Baseline Normalization

Similar to works [14,16], we performed for both datasets a baseline normalization procedure for the ECG signal recordings of each participant. In particular, for each subject we considered the ECG signal labeled as ’low stress’ as the baseline, and  subtracted each mean from all other segments of the subject. The objective of this procedure was to help reduce the individual bias, introduced in the measurements as a result of the difference in age, posture, level of physical conditioning, breathing frequency, and other factors. The pseudo-code of Algorithm 1 used for the baseline normalization is provided below:
**Algorithm 1** Baseline Normalization i←subjects_number **while**
i≠0
**do**     s←subjects[i]     m←mean(s[0])  ▹ At index 0 the ECG signal labeled as ‘low stress’ is accessed.     **for** l∈ecg_labels **do**         s[l]=s[l]−m     **end for**     i←i−1 **end while**


### 3.2. Dataset Augmentation

**Figure 3:**
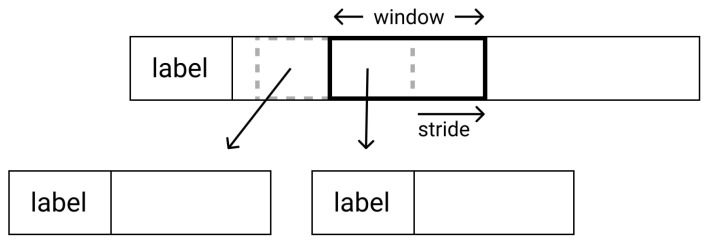
Sliding window dataset augmentation.

Due to the limited number of subjects, both datasets are relatively small for efficiently training a deep neural network. To generate more data for the training sets, we tested a data augmentation method following a sliding window approach. Through this method, the training samples are generated from multiple window-sized crops of the initial 1D signal segment, using a pre-defined stride. Figure 3 displays the procedure schematically.

### 3.3. Stress Level Analysis Architectures

#### 3.3.1. VGG Inspired Architecture

**Figure 4:**
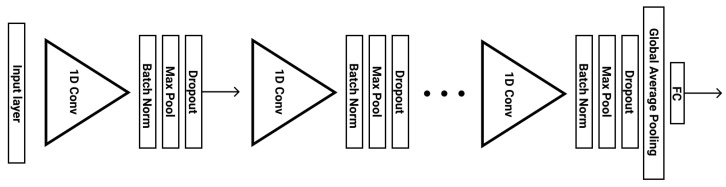
VGG inspired stress level analysis architecture.

VGG is a well-established CNN architecture that is widely used for efficient large-scale image recognition [43]. Inspired by this architecture, we employed a deep Convolutional Neural Network (CNN), which increases the number of channels, as the input dimensionality decreases in deeper layers. In particular, the number of channels starts from 64 and then increases by a factor of 2 at each stage, until it reaches 512. Our architecture includes 5 stages of 1D layers, with each stage consisting of the following layers: Convolution, Batch-Normalization, Activation, Max-Pooling and Drop-Out. The Batch Normalization layers to each stage, along with the Drop-Out and leaky RELU activation layers, which reduce overfitting and minimize the generalization error. After these 5 stages, a Global Average Pooling layer and a Fully-connected layer conclude the model. Figure 4 illustrates the VGG inspired stress level analysis architecture.

#### 3.3.2. Single 1D CNN Architecture

**Figure 5:**
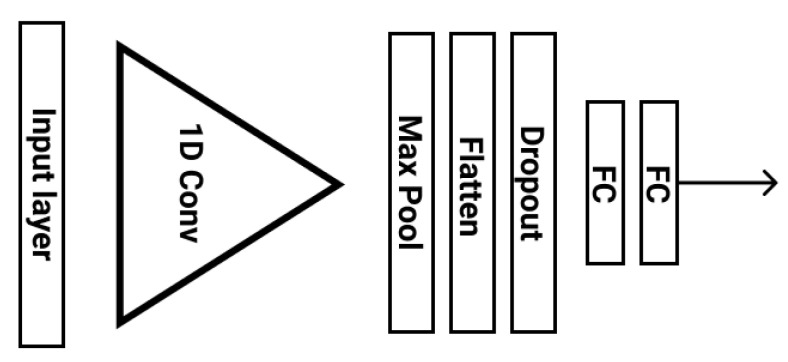
Single 1D CNN stress level analysis architecture.

This architecture is made up of one convolutional stage and two fully connected layers (Figure 5) along with a Drop-Out layer for regularization purposes. The key idea of this network is to set the lengths of the pooling and feature kernels to approximately span a period of the ECG signal, taking advantage of the correlation between the stress state and the morphology of the ECG signal RR interval. This was first observed by Hwang et al. and explained in detail in their paper [32]. Therefore, to utilize this correlation effectively we choose an appropriate pooling and feature length for our datasets. In particular, for a sampling frequency of 100 Hz and an average heart beat duration of around 0.8 s, the pooling length was set to span 80 raw ECG values. A pooling operation of that length is able to overcome the peak phase difference problem, since the features of a signal will eventually be extracted in a given pooling window regardless of the peak point. In addition, the feature length was set to 60, spanning all the important characteristics of the ECG signal (P, Q, R, S, and T waveforms) and without interfering with the next period of the signal. In case the frequency is 496, the pooling length becomes 400 and the feature length 300 ECG values.

A comparative advantage of this network to the VGG-inspired one, is that it can provide the required model capacity at a cost of just 28,866 parameters, as opposed to 1,554,819 parameters. This low model complexity reduces over-fitting and, at the same time, minimizes memory requirements, being deployable on low computational devices (e.g., the DARLENE wearable edge computing nodes, which consist of an AR HUD and a NVIDIA Jetson AGX Xavier micro-computer).

## 4. Results

Our models were trained and evaluated on the pre-processed datasets originating from the DriveDB and Arachnophobia datasets. To identify the best configuration for each task, we tuned our hyperparameters using 4-fold cross-validation. Then, we evaluated the models, trained using the best hyperparameters, on the test set. The train-validation-test split of the data was 60% for the train set, 20% for the validation set and 20% for the test set.

We adopted the approach followed by Seo et al. [33] and the partitioning into folds was carried out across subjects. More specifically, each fold can contain signal segments of different drivers, and each driver can have segments in multiple folds, having shuffled the collection of all driver segments before the split. Our hypothesis is that although the ECG signal waveform can differ from subject to subject, as mentioned by Ravenswaaij-Arts et al. [28], there might exist some global patterns—some features—of the ECG signal that can be used by our model to map the signal to the respective stress label.

Table 4 displays the average cross-validation accuracy of models trained on the DRIVEDB dataset, using different configurations with respect to the sampling frequency, sliding window (SW), number of classes, and architecture. In the case where the sliding window augmentation technique was employed (SW is Yes), the stride was 80 and 150, for frequencies 100 and 496, respectively.

As we can see in Table 4, our best-performing models for 2-level and 3-level stress classification, with average accuracies 98.3% and 85.1% respectively, were both trained using an ECG signal frequency of 496 Hz and employed the sliding window method. In the case of 2 classes, the VGG Inspired architecture led to the best accuracy, whereas in the case of 3 classes it was the single 1D CNN architecture. Furthermore, we can observe that in most cases, a higher sampling frequency positively affects the validation accuracy, with the exception of when we did not use the sliding window approach and the architecture was the single 1D CNN. Finally, it is evident that the sliding window technique leads to enhanced accuracy, in all cases.

When using the Arachnophobia dataset, the validation accuracy in both architectures decreases significantly. In particular, the average accuracy was 0.663 ± 0.013, using the VGG Inspired architecture and 0.698 ± 0.004, using the single 1D CNN architecture. The aforementioned results refer to the 3-level classification task and were acquired using 100 Hz ECG frequency and no sliding window. We attribute this reduced accuracy to the annotation assumption of the dataset, i.e., the normalized mean HR relates linearly with the stress level. This assumption might not always hold and, as a consequence, it causes the model to underfit. Provided the above, the Arachnophobia dataset was discarded from further experiments.

**Figure 6:**
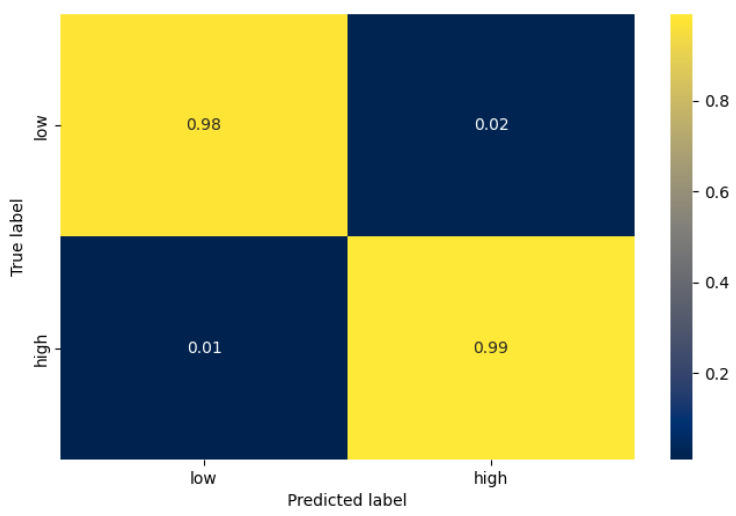
The confusion matrix of the best model for the stress detection task (VGG inspired architecture).

**Figure 7:**
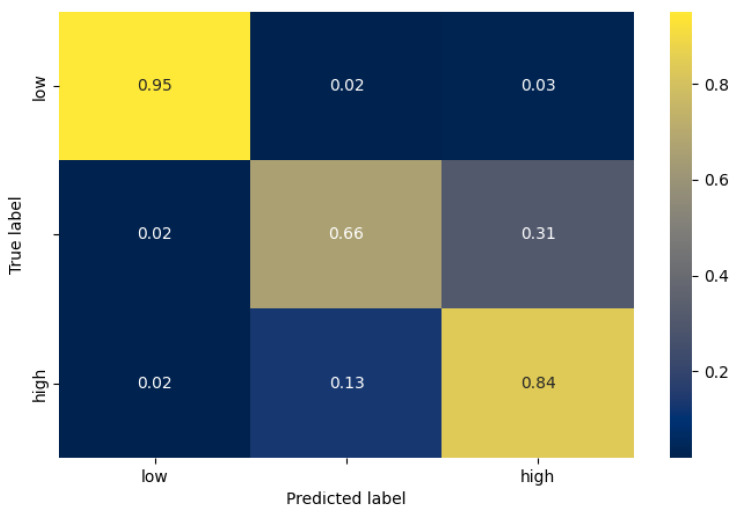
The confusion matrix of the best model for the 3-level stress classification task (single 1D CNN architecture).

Having identified which hyperparameters lead to increased accuracy, we used them to train and evaluate a model for each stress-classification task and each architecture. In Table 5, the accuracy of the test set is depicted for each case. Similar to the validation average accuracy, the VGG architecture performs best in the 2-class case, whereas the single 1D CNN in the 3-class case. In Figure 6 and Figure 7 we can see the confusion matrix of the best model for each task, while in Table 6, the number of samples of each class are mentioned. In the case of 2 classes (stress detection), we can see that the true positive and true negative rate is very high (0.99%, and 0.98% respectively), while the false negative rate is 2 times higher than the false positive rate. Furthermore, in the case of 3-level stress classification, we can observe that, as expected, the model can differentiate with higher accuracy between the low and high classes, than between the moderate and high classes. More specifically, the highest miss-classification rate occurs when the true label is moderate (33% in total), where the model predicts a high stress level at 31%.

## 5. Discussion

### 5.1. Comparison with Previous Studies

Although accuracy is not the most informative metric, it is the most common across related literature and thus it is the one we use to compare our results with those of different approaches. We first compare our deep architectures’ performance on the 3-level classification problem, with that of other two deep architectures, which were evaluated on the DriveDB dataset in the work of Seo et al. [33]. These models are the state-of-the-art DNN models on the DriveDB dataset, and among the best-performing for 2- and 3-class stress classification, using heart related features. In Table 7, we can see that our models’ accuracies of 83.09% and 83.55% surpass that of the other models, while using a smaller time window as input for making a prediction, significantly reducing the amount of necessary memory resources. More specifically, our approach uses a time window of 3 s, which corresponds to 1488 signal samples, in comparison to the 24,800 of other approaches. Moreover, our approach achieves its accuracy while utilizing only one signal, the ECG signal, in comparison to [33], which also uses RSP. Finally, our reported accuracy is the test set accuracy, while the other accuracies are derived as the average validation accuracy of 5-fold cross validation [33].

Finally, in Table 8, we compare our results with those of other works, which use heart related features to classify the stress state, independently of the evaluation dataset choice (see Table 8). Regarding the 2-level stress classification, we can observe that our best-performing model achieves state-of-the-art accuracy, while using a 3 s time window instead of 10 s, and automatically learned representations instead of handcrafted ones. With respect to the 3-level stress classification, our best-performing model exhibits a margin of 9.25% from the works of [34,44]. Factors that lead to this difference are the fact that [44] leverages multiple signals and [34] uses a much wider time window of 25 s, as well as the fact that their models are being trained and evaluated on other (non-public) datasets.

### 5.2. Model Capacity

In this work we have implemented two divergent architectures in terms of the capacity of the model. In particular, the single 1D CNN architecture has just 28,866 parameters as opposed to the 1,554,819 parameters of the VGG-inspired one. In spite of it being more lightweight, having considerably less parameters, our results indicate that the single 1D CNN can achieve comparative accuracy for our classification task, even surpassing the VGG-inspired architecture, in the more difficult 3-level stress classification. Considering its combination of high accuracy and low resources allocation, it could be efficiently deployed in mobile applications as well as wearable devices (e.g., the DARLENE wearable edge computing nodes).

### 5.3. Limitations and Future Work

Although we have acquired state-of-the-art results, in terms of accuracy, in the 2- and 3-level stress classification task, using ultra-short samples of raw ECG signal, there were some practical obstacles that we had to confront. These were the limited task-related open datasets available for training deep learning models and the fact that only a small minority of them provide annotations for more than two classes of stress. The existence of datasets providing multilevel stress annotations would allow for more accuracy and useful feedback in practical domains, as mentioned by Ahmad et al. [49]. Furthermore, the open-available stress related datasets are of limited size, hindering the effective evaluation of developed models. Finally, another inherent limitation of such a Deep Learning approach is that the model is a “black box”, providing no indication to the exact physiological process that contributes to quantifying the level of stress.

To confront the aforementioned dataset limitations, we aim to create our own dataset, which will be specific to our application domain, namely stress experienced by emergency operators and LEAs during their work. This way we can train and evaluate our proposed architectures more effectively, and on more representative target domain data. By collecting a profusion of data from different individuals, we will investigate domain-adaptation techniques, for the purpose of personalizing the stress-level predictions. Moreover, we plan to utilize multiple physiological signals, such as EDA and RSP, and investigate whether and which fusion leads to better performance. To gain a better understanding of the learned representations, we also plan to apply different visualization approaches to our models. This way, we could explore whether the trained convolution layer generates feature patterns specific to the stress classification task.

## 6. Conclusions

Throughout this work, our aim was to design and implement CNN models that are able to assess the user stress level through raw ECG signals. With that ability, it could provide useful feedback to context-aware and personalised applications concerning emergency operators and in particular LEAs, who can wield wearable devices of low computational capacity. Our proposed architectures—one being simple and with low memory footprint, and the other being deeper with greater model capacity—met that goal by yielding state-of-the-art accuracy on the DriveDB dataset both for the binary and the multi-class stress classification task. Specifically, our architectures and data processing techniques reported an accuracy of 98.77% and 83.55%, respectively. Finally, we explored two open-access datasets and provided insights regarding their problems and limitations. 

## Figures and Tables

**Table 1:** DriveDB observations and errors.

Subject	Used in This Work	Comments/Observations
drive01	NO	No marker signal is provided.
drive02	NO	The marker signal has more than 8 peaks.
drive03	NO	No marker signal is provided.
drive04	NO	The peaks of the marker signal are not distinguishable.
drive05	PARTIALLY	We discarded the first two events(invalid signal values).
drive06	YES	N/A
drive07	YES	N/A
drive08	YES	N/A
drive09	NO	The marker signal has less than 8 peaks.
drive10	YES	N/A
drive11	YES	N/A
drive12	NO	Missing ECG signal data.
drive13	YES	N/A
drive14	YES	N/A
drive15	YES	N/A
drive16	NO	The marker signal has less than 8 peaks.
drive17	NO	Was not used because it is split in two parts.

**Table 2:** DriveDB driver events and corresponding annotations for 2 and 3 classes of stress.

Classes	Initial Rest	City1	Hwy1	City2	Hwy2	City3	Final Rest
2	NO	YES	YES	YES	YES	YES	NO
3	LOW	HIGH	MODERATE	HIGH	MODERATE	HIGH	LOW

**Table 3:** Number of samples per dataset, using a window size of 3 s.

Dataset	Low	Moderate	High	Total
DriveDB	1800 (29.51%)	1700 (27.87%)	2600 (42.62%)	6100
Arachnophobia	5507 (23.67%)	8882 (38.17%)	8881 (38.17%)	23270

**Table 4:** Models’ cross-validation accuracy using different configurations.

Frequency (Hz)	SW	Classes	VGG Inspired	Single 1D CNN
100	No	2	0.939 ± 0.024	0.950 ± 0.012
100	No	3	0.764 ± 0.043	0.803 ± 0.009
100	Yes	2	0.963 ± 0.024	0.959 ± 0.018
100	Yes	3	0.804 ± 0.006	0.823 ± 0.008
496	No	2	0.972 ± 0.009	0.943 ± 0.019
496	No	3	0.802 ± 0.022	0.796 ± 0.023
496	Yes	2	**0.983 ± 0.004**	0.960 ± 0.008
496	Yes	3	0.822 ± 0.029	**0.851 ± 0.016**

**Table 5:** Test set accuracy for each stress-classification task and architecture.

Classes	VGG Inspired	Single 1D CNN
2	98.77%	95.66%
3	83.09%	83.55%

**Table 6:** Number of samples per class in test set of best models.

Class	Samples	Percentage
LOW	460	30.26%
MODERATE	340	22.37%
HIGH	720	47.37%

**Table 7:** Stress classification task with 3 stress labels (low, moderate, high).

Models	DeepERNet	DeepECGNet	Single 1D Conv.	VGG Insp.
Accuracy (%)	83.0	75.0	83.55	83.09
Window	24,800	24,800	1488	1488
Frequency (Hz)	496	496	496	496
Time (sec)	50	50	3	3
Augmentation	no	no	yes	yes
Signals	ECG & RSP	ECG	ECG	ECG

For DeepERNet refer to [33] and for DeepECGNet refer to [32].

**Table 8:** Stress classification using heart related features.

Method	Accuracy (%)	Method	Data	Window Size	Classes
VGG insp.	98.77	CNN	ECG	3 s	2
[45]	98.69	CNN	HRF	10 s	2
[36]	98.3	CNN-LSTM	ECG	-	2
[46]	95.67	CNN	HR and other	30 s	2
[47]	90.19	CNN	ECG	10 s	2
[16]	89.8	CNN	ECG	60 s	2
[32]	87.39	CNN-RNN	ECG	10 s	2
[33]	83.9	CNN	ECG and RSP	50 s	2
[31]	82.7	CNN	ECG	10 s	2
[44]	92.8	CNN-LSTM	ECG and other	5 s	3
[34]	92.8	CNN	ECG	25 s	3
[48]	86.5	CNN-BiLSTM	ECG	10 s	3
single 1D Conv.	83.55	CNN	ECG	3 s	3
[33]	83.0	CNN	ECG and RSP	50 s	3
[49]	85.45	CNN	ECG	30 s	5

## Data Availability

Not applicable.
